# Effect of Glazing Dwell Time and Cooling Duration on Flexural Strength and Surface Microhardness of 3 mol% Yttria‐Stabilized Tetragonal Zirconia: An In Vitro Study

**DOI:** 10.1111/jerd.70232

**Published:** 2026-07-14

**Authors:** Shubha Sarmalkar, Aradhana Nagarsekar, Meena A. Aras, Umesh Y. Pai, Ridhima Gaunkar, Prajakta R. Volvaikar

**Affiliations:** ^1^ Department of Prosthodontics Goa Dental College and Hospital Bambolim India; ^2^ Department of Prosthodontics Manipal College of Dental Sciences Mangalore, Manipal Academy of Higher Education Manipal India; ^3^ Department of Public Health Dentistry Goa Dental College and Hospital Bambolim India

**Keywords:** cooling protocol, esthetic dentistry, flexural strength, glazing, monolithic zirconia, surface microhardness, Weibull analysis

## Abstract

**Objective:**

To evaluate the effect of glazing dwell time and cooling duration on the flexural strength and surface microhardness of high‐translucency 3 mol% yttria‐stabilized tetragonal zirconia polycrystal (3Y‐TZP).

**Materials and Methods:**

Total of 108 rectangular zirconia specimens (20 × 4 × 2 mm) were fabricated from preshaded monolithic blocks and sintered at 1530°C. Specimens were divided into nine groups (*n* = 12). The control group (NG) remained unglazed. Experimental groups underwent conventional firing (CF;1‐min dwell) or extended firing (EF; 3‐min dwell) at 850°C, each combined with four cooling durations (4,7,9, or 12 min) between 850°C and 600°C. Flexural strength was evaluated using a four‐point bending test (ISO 6872:2015) with Weibull analysis, while surface microhardness was measured using the Vickers method (50 g, 10 s). Data were analyzed using two‐way ANOVA and Tukey's HSD test (*α* = 0.05).

**Results:**

Significant differences were observed in flexural strength and microhardness (*p* < 0.001). The unglazed group showed the highest strength (698 ± 18 MPa; *m* = 46). Among glazed groups, EF3 (3‐min dwell/9‐min cooling) exhibited the highest characteristic strength (*σ*₀ = 657 MPa). EF1 (3‐min dwell/4‐min cooling) showed the lowest strength (466 ± 23 MPa), below ISO requirements. Microhardness decreased with longer cooling durations.

**Conclusions:**

Glazing parameters significantly affect 3Y‐TZP properties. Extended firing with controlled slow cooling (9 min) optimizes strength and reliability, while rapid cooling compromises performance.

## 1 Introduction

Esthetic excellence and mechanical reliability are no longer competing priorities in restorative dentistry; they have become simultaneous and nonnegotiable clinical expectations. High‐translucency 3 mol% yttria‐stabilized tetragonal zirconia polycrystal (3Y‐TZP) has emerged as the material best positioned to meet both demands, combining fracture resistance that supports posterior load‐bearing applications with optical properties that increasingly approximate the depth and translucency of natural dentition [1, 2]. As monolithic zirconia restorations continue to replace veneered frameworks in everyday clinical practice, the processing decisions made in the dental laboratory have become the primary determinants of both structural integrity and esthetic outcome.

The glazing procedure in monolithic zirconia fabrication serves a dual and clinically inseparable function. Optically, the glaze layer establishes the surface texture, specular gloss, and light scattering properties that govern the restoration's visual harmony with natural dentition [1, 3]. Structurally, it occludes surface microcracks and machining artifacts introduced during CAD/CAM milling, limiting sites of potential crack initiation [3]. Both functions are contingent on the thermal parameters applied conventionally at 850°C–900°C with dwell times of 1–2 min and cooling to 600°C over 3–4 min, and both are therefore vulnerable to the residual stresses and phase alterations that any additional thermal cycle introduces into the zirconia matrix.

The cooling phase following glaze firing is of particular interest, as thermal gradients generated during cooling are known to induce residual stresses within ceramic materials. In glass ceramic systems, extended slow cooling has been associated with improved flexural strength and reduced surface hardness, a reduction that carries clinical value by diminishing the abrasive potential of restorations against opposing enamel [4, 5, 6]. However, zirconia is a polycrystalline material governed by transformation toughening mechanics entirely distinct from glass ceramic behavior; the implications of cooling rate for its phase stability, residual stress distribution, and surface properties cannot be inferred by analogy and require direct investigation [7, 8, 9].

The evidence base for zirconia specific glazing optimization remains critically limited. The only available data on cooling duration demonstrated that extending cooling to 7 min improved flexural strength over the standard 4‐min protocol, yet values remained below those of unglazed specimens, suggesting that the interaction between dwell time and cooling rate may be more complex than a single variable modification [10]. The effect of extended glazing dwell time on zirconia, a parameter shown to promote crack healing in glass ceramics at 11–15 min has not been investigated for polycrystalline zirconia systems, leaving a substantive and clinically relevant gap in the literature [4].

For the clinician, this knowledge gap has a direct practical consequence: glazing protocols are currently applied to monolithic zirconia restorations based on manufacturer defaults or glass ceramic precedent, without an evidence base specific to the material being fired. If suboptimal thermal parameters are systematically compromising the mechanical performance of glazed zirconia restorations, the clinical implications such as accelerated failure and reduced restoration longevity are significant and preventable. The present study therefore aimed to evaluate the effect of extended glazing dwell time (3 min) and controlled slow cooling durations (up to 12 min) on the flexural strength and surface microhardness of high translucency monolithic 3Y‐TZP, with the goal of providing an evidence‐based foundation for optimizing glazing protocols in clinical laboratory practice. The research hypothesis stated that variations in glazing dwell time and cooling duration significantly influence the flexural strength and surface microhardness of high‐translucency 3Y‐TZP.

## 2 Materials and Methods

### 2.1 Sample Size Determination

The sample size of *n* = 12 per group was determined a priori using GPower software (Version 3.1). Based on a two‐way analysis of variance (ANOVA) design with nine groups, an anticipated effect size of *f* = 0.40 (large effect, derived from pilot study), a significance level of *α* = 0.05, and a desired statistical power of 0.80 (80%), the minimum required sample size was calculated as nine specimens per group. The sample size was increased to *n* = 12 per group to account for potential specimen fractures during preparation.

### 2.2 Specimen Preparation

A total of 108 rectangular zirconia specimens were fabricated with preshaded A2 high‐translucency ST zirconia blank (Upcera Dental Technology, Shenzhen, China), with a reported translucency of 43%, flexural strength of 1300 MPa, and a yttria content of 3 mol% (3Y‐TZP), using a five‐axis milling unit (ARUM 5X‐450, Doowon, Korea). Specimens were designed with dimensions of 25 × 5 × 2.5 mm to yield final sintered dimensions of 20 × 4 × 2 mm following the anticipated 20% sintering shrinkage, in accordance with ISO 6872:2015 for flexural strength testing. Sintering was performed at 1530°C for 2 h in a dedicated furnace (Zircom, KDF, Japan). Postsintering dimensions were verified with a digital vernier caliper (Mitutoyo Corporation, Japan).

### 2.3 Experimental Grouping and Glazing Procedures

Specimens were randomly assigned to nine groups (*n* = 12 per group). Group NG served as the unglazed control. Glazing was performed on groups conventional firing (CF) and extended firing (EF) using a thin uniform application of overglaze paste (Universal Overglaze, Dentsply Sirona, USA) fired in a ceramic furnace (Programat P310, Ivoclar Vivadent, Liechtenstein). Two firing protocols were evaluated: CF at 850°C with a 1‐min dwell time, and EF at 850°C with a 3‐min dwell time. Each protocol was combined with four cooling durations from 850°C to 600°C: 4 min (manufacturer‐recommended rapid cooling), 7, 9, or 12 min. Subgroups were designated CF1–CF4 and EF1–EF4 accordingly. All specimens were bench‐cooled to ambient temperature following the 600°C threshold and stored in sealed containers for 12 h prior to mechanical testing. A schematic representation of the study design is presented in Figure 1.

**FIGURE 1:**
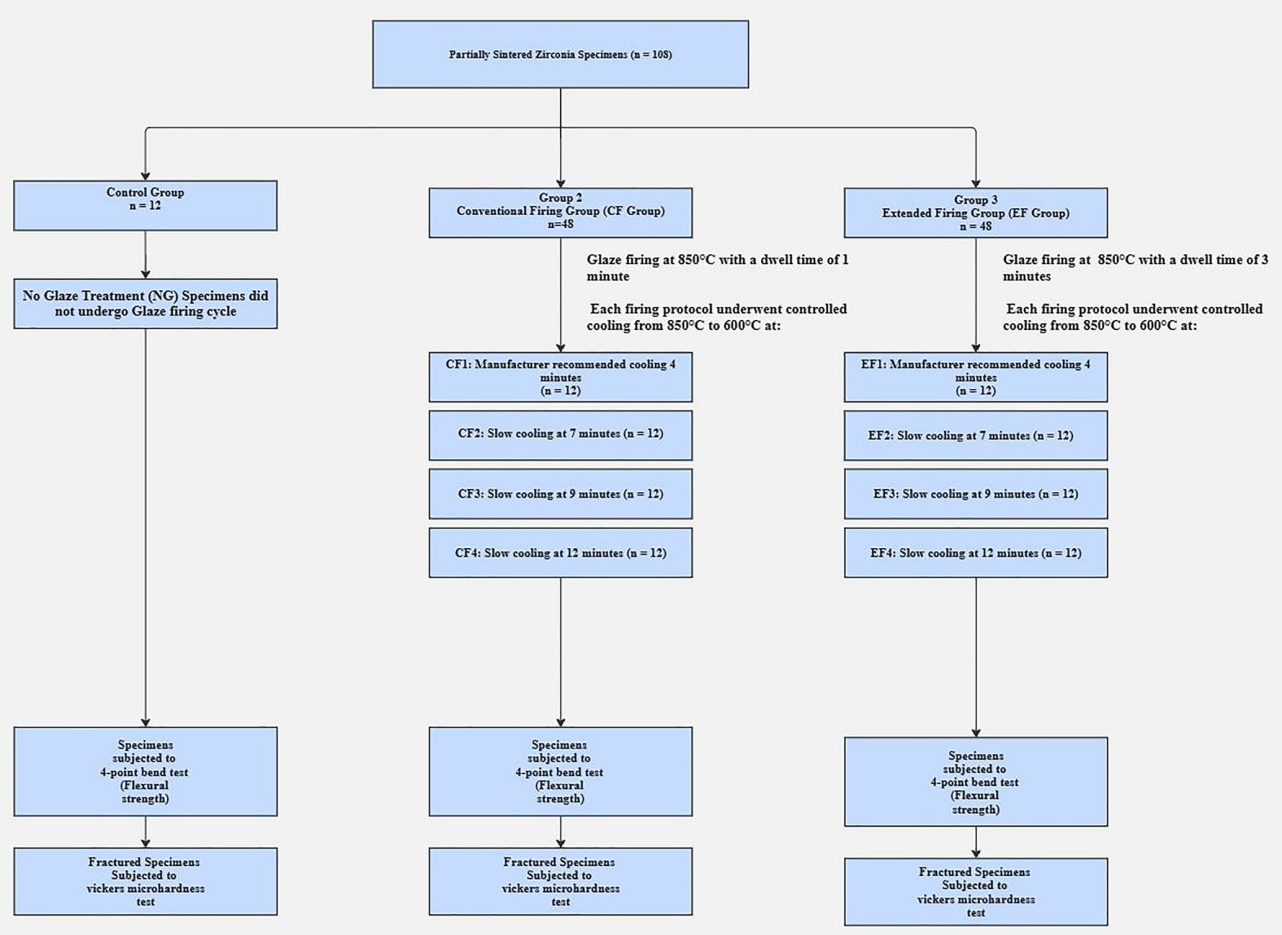
Schematic overview of the study design.

### 2.4 Flexural Strength Testing

Flexural strength was assessed using a universal testing machine (UNITEST‐10, ACME Engineers, India) with a customized four‐point bending fixture in compliance with ISO 6872:2015. Each specimen was positioned with the glazed surface in tension at a crosshead speed of 1.0 mm/min until fracture. Flexural strength (*σ*) was calculated as follows: *σ* = 3*PL*/(4*wb* ^2^), where *P* is the fracture load (N), *L* is the outer support span (mm), *w* is specimen width (mm), and *b* is specimen thickness (mm).

### 2.5 Weibull Analysis

Two‐parameter Weibull analysis was performed to characterize the distribution and reliability of flexural strength data. The Weibull modulus (*m*) and characteristic strength (*σ*₀) were determined by both median rank regression and maximum likelihood estimation using Stata Release 18 (StataCorp LLC, Texas, USA). The failure probability was expressed as: *Pf* = 1 − exp[−(*σ*/*σ*₀)^*m*^], where *Pf* was estimated using the median rank approximation (rank −0.5)/*N*.

### 2.6 Surface Microhardness

Following flexural testing, fractured specimens were subjected to microhardness analysis. Vickers hardness was measured using a microhardness tester (Reichert, Vienna, Austria) with a 50 g load applied for 10 s. Indentations were made on glazed external surfaces using a diamond indenter with an apex angle of 136°. Vickers hardness numbers (VHN) were recorded for each sample.

### 2.7 Statistical Analysis

Statistical analyses were performed using IBM SPSS Statistics v.22 (IBM Corporation, New York, USA). Data normality was confirmed using the Shapiro–Wilk test. Intergroup differences in flexural strength and surface microhardness were analyzed using two‐way ANOVA to evaluate the effects of dwell time (two groups), cooling duration (four subgroups), and their interaction, with Tukey's HSD post hoc test applied for pairwise comparisons. Weibull analysis was performed to assess the reliability and predictability of flexural strength distribution, with Weibull modulus (*m*) and characteristic strength (*σ*₀) determined by both median rank regression and maximum likelihood estimation. Statistical significance was set at *p* < 0.05.

## 3 Results

As per two‐way ANOVA, there was a significant interaction between dwell time and cooling rate for both flexural strength and surface microhardness, indicating that the mechanical response to postfiring cooling period was dependent on the dwell time employed. Therefore, pairwise comparisons were performed using Tukey's HSD post hoc test. The results are presented in Table 1, with superscript letters serving as statistical grouping indicators. Groups sharing at least one common letter were not significantly different, whereas groups with no common letters differed significantly (*p* < 0.05). Letter assignments were made independently for flexural strength, load, and surface microhardness.

**TABLE 1:** Descriptive statistics (mean ± SD) and two‐way ANOVA results for load at fracture, flexural strength, and Vickers microhardness of partially sintered zirconia specimens subjected to conventional firing (CF) and extended firing (EF) protocols at varying cooling times.

Group	Cooling time (min)	Load (N)		Flexural strength (MPa)		Microhardness (HV)	
		Mean	SD	Mean	SD	Mean	SD
CF	Prefiring (0)	822^hi^	110	698^i^	18	1243^a^	64
	4 (CF1)	530^ab^	60	504^b^	18	1236^ah^	75
	7 (CF2)	577^abc^	41	515^bc^	27	1179^begh^	41
	9 (CF3)	729^fg^	27	629^g^	26	1054^fg^	38
	12 (CF4)	630^cde^	24	554^de^	33	986^ce^	20
EF	Prefiring (0)	822^hij^	110	698^ij^	18	1243^a^	64
	4 (EF1)	512^a^	16	466^a^	23	998^cf^	34
	7 (EF2)	602^bcd^	14	534^bcd^	29	960^cd^	23
	9 (EF3)	747^gh^	60	643^gh^	32	928^bde^	57
	12 (EF4)	662^def^	19	579^ef^	23	916^bd^	24

Two‐way ANOVA			
Source of variation	Load *F* (df)	Flexural strength *F* (df)	Microhardness *F* (df)
Group (firing protocol)	**97 (4)** ***	**275 (4)** ***	**138 (4)** ***
Cooling time	1 (1)	1 (1)	**224 (1)** ***
Group × cooling Time	1 (4)	**6 (4)** *	**26 (4)** ***

*Note:* Bold values indicate statistically significant differences (*p* < 0.05). Values are presented as mean (SD). Superscript letters indicate Tukey's HSD statistical groupings.

* *p* < 0.05.

*** *p* < 0.001.

### 3.1 Flexural Strength

All glazed specimens exhibited significantly reduced flexural strength compared with the unglazed control (all *p* < 0.001; Table 1).

#### 3.1.1 CF

CF3 (629 ± 26 MPa) recorded the highest flexural strength within the CF protocol, significantly exceeding CF1 (+125 MPa, *p* < 0.001), CF2 (+114 MPa, *p* < 0.001), and CF4 (+74 MPa, *p* < 0.001). CF1 and CF2 did not differ significantly from each other (*p* > 0.05), indicating equivalent mechanical performance at the two shortest conventional (rapid) cooling intervals.

#### 3.1.2 EF

EF3 (643 ± 32 MPa) exhibited the highest flexural strength within the EF protocol and was significantly greater than EF1 (+176 MPa, *p* < 0.001) and EF2 (+108 MPa, *p* < 0.001). EF4 (579 ± 23 MPa) occupied an intermediate position, higher flexural strength than both EF1 and EF2, yet remaining significantly below EF3, consistent with a progressive strength gain that peaks at 9 min and attenuates beyond it. Unlike the CF protocol, EF1 and EF2 differed significantly from each other (+88 MPa, *p* < 0.001), reflecting a steeper early strength trajectory under EF and suggesting greater sensitivity to the 4–7‐min cooling period.

#### 3.1.3 Between Protocol Comparisons

The greatest reductions in flexural strengths were observed in the shortest 4‐min cooling interval under both protocols. Conversely, the 9‐min cooling interval produced the smallest deviation from control under both protocols CF3 and EF3 (Δ = −14 MPa, *p* > 0.05), underscoring a progressive, cooling duration dependent recovery of flexural strength.

### 3.2 Weibull Analysis

Weibull analysis confirmed the superior mechanical reliability of the NG group, which exhibited the highest Weibull modulus (*m* = 46 [39–53]) and characteristic strength (*σ*₀ = 706 MPa [704–709]). Within the experimental subgroups, EF3 demonstrated the highest characteristic strength (*σ*₀ = 657 MPa [649–666]), while EF4 exhibited a comparatively higher Weibull modulus (*m* = 29 [24–34]), indicating greater structural reliability. CF3 yielded a favorable combination of characteristic strength (*σ*₀ = 641 MPa) and reliability (*m* = 27). The lowest characteristic strengths were recorded for EF1 (*σ*₀ = 477 MPa) and CF1 (*σ*₀ = 512 MPa), with rapid cooling consistently reducing both strength and predictability of failure. The reliability of flexural strength, as determined by Weibull modulus (*m*) and characteristic strength (*σ*₀), is summarized in Table 2. Weibull probability plots are presented in Figure 2.

**TABLE 2:** Weibull modulus (*m*) characteristic strength (*σ*₀) values indicating reliability of flexural strength.

Group	Mean flexural strength (MPa) (SD)	Weibull modulus (*m*) (95% CI)	Characteristic strength *σ*_0_ (MPa) (95% CI)
CF1	504 (18)	32 (25–39)	512 (508–516)
CF2	515 (27)	22 (17–27)	527.17 (521–534)
CF3	629 (26)	27 (21–33)	641 (634–648)
CF4	554 (33)	19 (15–24)	570 (561–578)
Control	698 (18)	46 (39–53)	706 (704–709)
EF1	466 (23)	23 (17–29)	477 (470–484)
EF2	534 (29)	21 (15–27)	548 (539–556)
EF3	643 (32)	23 (18–28)	657 (649–666)
EF4	579 (23)	29 (24–34)	589 (585–594)

**FIGURE 2:**
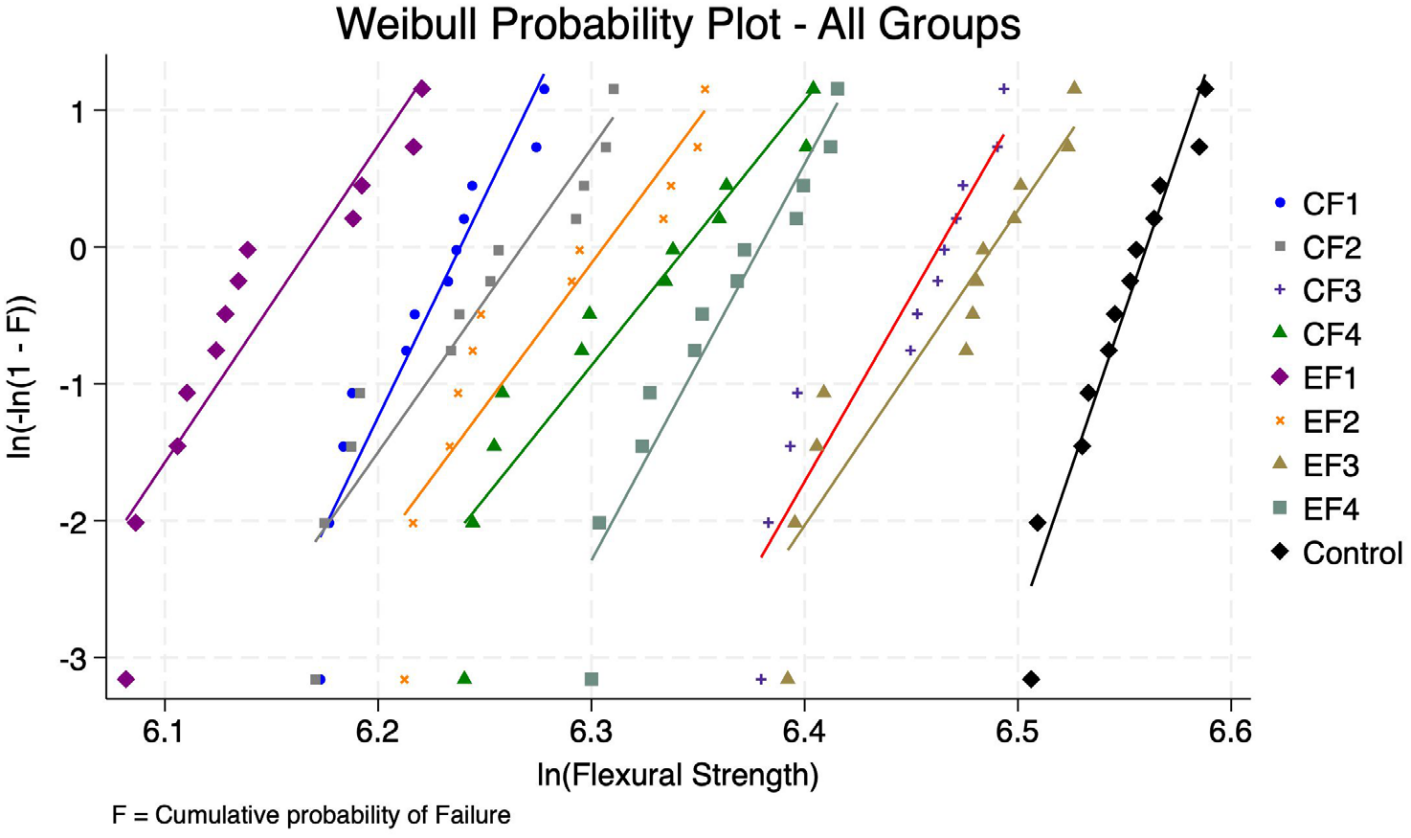
Graphical representation of Weibull distribution analysis based on probability of failure.

### 3.3 Surface Microhardness

All glazed specimens exhibited significantly reduced surface microhardness compared with the unglazed control (all *p* < 0.001; Table 1).

A graphical comparison of mean surface microhardness values (VHN) among all groups and subgroups is illustrated in Figure 3.

**FIGURE 3:**
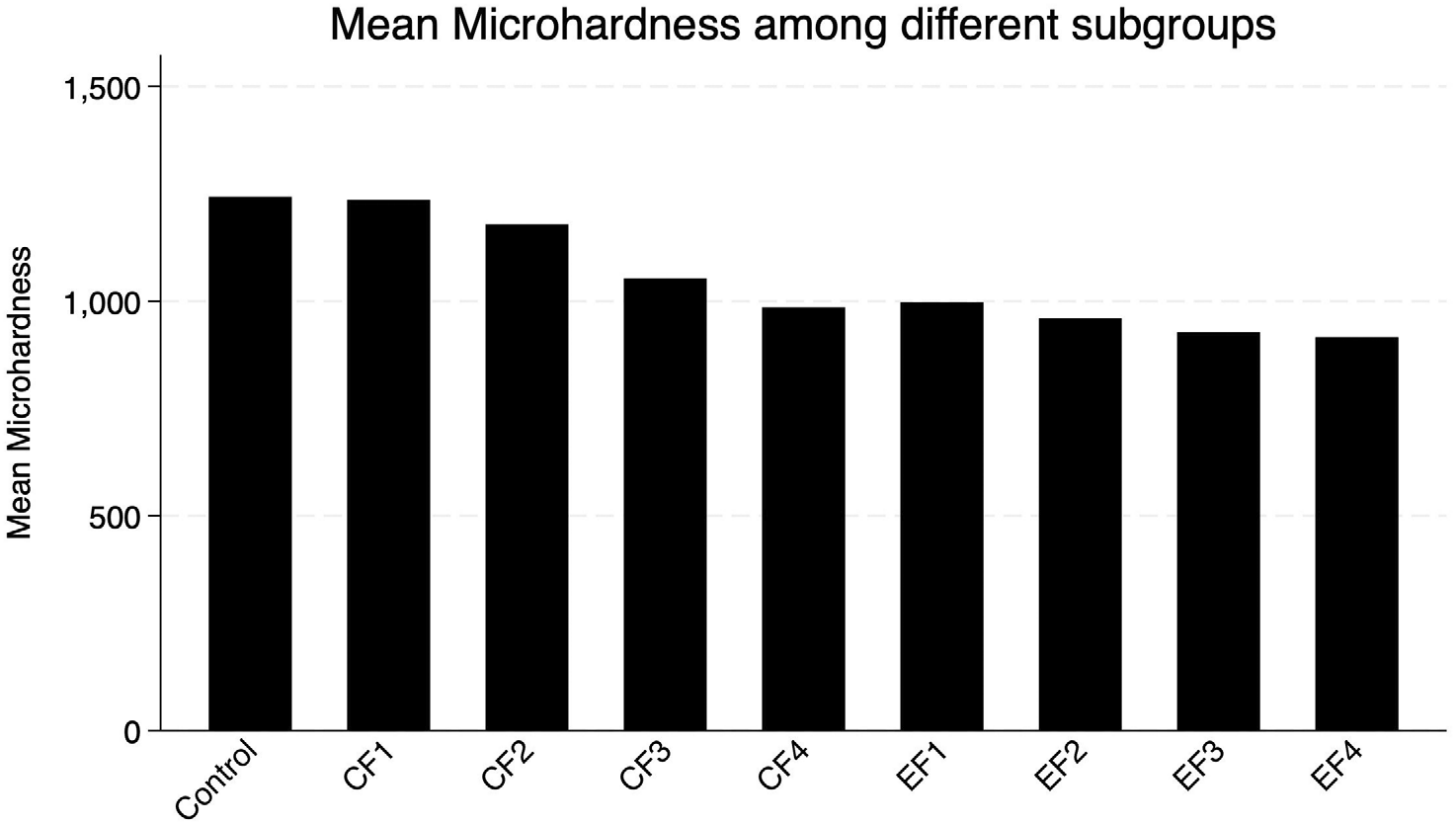
Comparison of mean surface microhardness values (Vickers hardness number, VHN) among all groups and subgroups.

#### 3.3.1 CF

Within the CF protocol, microhardness declined progressively with increasing cooling duration. CF1 was significantly greater than CF3 (−182 HV, *p* < 0.001) and CF4 (−250 HV, *p* < 0.001). CF1 and CF2 (1179 HV) did not differ significantly (*p* > 0.05), nor did CF2 and CF3, suggesting a transitional plateau in the mid‐cooling range. CF3 (1054 HV) and CF4 (986 HV) differed significantly (68 HV, *p* < 0.05), confirming a hardness decline beyond 9 min under CF. CF1 (1236 ± 75 HV) was the only postfiring group that did not differ significantly from the unglazed control (Δ = 8 HV, *p* > 0.05), demonstrating that CF followed by a 4‐min cooling interval uniquely preserved surface hardness.

#### 3.3.2 EF Groups

The EF protocol exhibited an interesting microhardness plateau beyond the 7‐min cooling interval. EF1 (998 HV) demonstrated the highest microhardness. EF2 (960 HV), EF3 (928 HV), and EF4 (916 HV) did not differ significantly from one another (all *p* > 0.05). This plateau pattern (unlike progressive CF decline) provides the mechanistic basis for the significant firing dwell time and cooling rate interaction for microhardness.

#### 3.3.3 Between Protocol Comparisons

CF was significantly superior to EF in surface microhardness at every matched cooling interval: CF1 versus EF1 (+238 HV), CF2 versus EF2 (+219 HV), CF3 versus EF3 (+126 HV), and CF4 versus EF4 (+70 HV; all *p* ≤ 0.05).

## 4 Discussion

Glazing represents the intersection of esthetics and engineering in monolithic zirconia fabrication. The present study demonstrated that both glazing dwell time and cooling duration significantly influence the flexural strength and surface microhardness of high translucency 3Y‐TZP, thereby supporting the research hypothesis. These findings carry direct implications for clinical laboratories seeking to optimize glazing protocols without compromising the structural durability that underpins long‐term esthetic success.

The unglazed control exhibited the highest flexural strength, showing that any additional thermal cycle introduces residual stresses that diminish mechanical performance. Nevertheless, the performance deficit was substantially reduced by implementing extended dwell times combined with controlled slow cooling. EF3 (3‐min dwell/9‐min cooling) approached control level strength among all experimental subgroups, suggesting that an optimal glazing protocol largely mitigates the deleterious effects of thermal cycling.

The improvement associated with slow cooling is consistent with the transformation toughening mechanism intrinsic to Y‐TZP. Gradual thermal relaxation is understood to preserve the metastable tetragonal phase and its associated compressive surface stresses, inhibiting crack propagation through stress‐induced tetragonal‐to‐monoclinic transformation accompanied by approximately 3%–5% volumetric expansion. Conversely, rapid cooling introduces thermal shock related surface microcracks and residual tensile stresses that counteract this toughening behavior. The detrimental impact of rapid cooling was most pronounced following EF: EF1 produced the lowest characteristic strength and was the only group to fall below the 500 MPa ISO threshold, underscoring that EF without slow cooling is contraindicated in clinical laboratory practice.

The Weibull modulus provides an important complementary metric to mean strength by quantifying the homogeneity of failure behavior. EF4, despite showing slightly lower mean strength than EF3, demonstrated a higher Weibull modulus, suggesting that prolonged cooling improves structural homogeneity even at the cost of minor strength reduction. CF3 similarly exhibited a favorable balance of strength and reliability. In contrast, rapid cooling groups (CF1, EF1) showed lower characteristic strengths, confirming that thermal shock introduces not only weaker but also less predictable failure behavior, a clinically important distinction for load‐bearing restorations.

Surface microhardness decreased progressively with extended cooling, consistent with the Hall–Petch relationship: prolonged thermal exposure promotes grain coarsening, which reduces resistance to indentation. From a clinical perspective, although a reduction in hardness may theoretically decrease the wear resistance of the restoration, it may also reduce antagonist enamel abrasion. This is particularly relevant given that natural enamel hardness ranges between 294 and 408 VHN, whereas unmodified zirconia typically exhibits values of 900–1200 VHN. Moderate hardness reductions achieved through controlled slow cooling may therefore improve biological compatibility by minimizing wear of opposing dentition without compromising structural function above ISO mandated thresholds.

From an esthetic standpoint, the glazing protocol exerts influence beyond the mechanical domain. The glaze layer is the primary determinant of the surface texture, gloss, and light reflective quality of a monolithic zirconia restoration, properties that define its optical integration with adjacent natural dentition. The glaze surface characteristics, including smoothness and gloss retention, are sensitive to firing temperature and duration. Although surface gloss and roughness were not directly measured in the present study, the controlled thermal conditions associated with slow cooling are expected to produce a more homogeneous glaze surface by allowing adequate flow and leveling of the glaze material before vitrification.

Furthermore, the reduction in surface microhardness observed with slow cooled protocols may contribute to a glaze surface that is less susceptible to microchipping under functional loading, preserving surface gloss over time. These esthetic considerations reinforce the clinical rationale for adopting slow cooling protocols and highlight the need for future studies to directly characterize the optical and surface texture outcomes of different glazing conditions alongside mechanical endpoints.

The use of four‐point bending in accordance with ISO 6872:2015 rather than biaxial flexure is a methodological strength, as it applies a uniform stress distribution over a larger specimen area and increases sensitivity to surface initiated flaws, providing a clinically representative assessment of ceramic failure behavior. Summary of the principal findings of the study and their potential clinical relevance are presented in Table 3.

**TABLE 3:** Summary of the principal findings of the study and their potential clinical relevance.

Variable	Key findings	Mechanical insight	Clinical implications
EF3 (extended firing + 9‐min slow cooling)	Highest Weibull characteristic strength (*σ*₀ = 657 MPa); high reliability (*m* = 23)	Preservation of tetragonal‐to‐monoclinic (t → m) transformation toughening; reduced residual thermal stresses; limited grain growth.	Recommended for high‐load clinical scenarios such as posterior fixed dental prostheses (FDPs)
EF4 (extended firing + 12‐min slow cooling)	High Weibull characteristic strength (*σ*₀ = 589 MPa); highest reliability (*m* = 29)	Enhanced predictability due to thermal stress mitigation, slight increase in grain size without compromising strength.	Suitable for moderate‐load scenarios where reliability is prioritized
EF1 (extended firing + rapid cooling/manufacturer‐ recommended 4 min)	Reduced Weibull characteristic strength (*σ*₀ = 477 MPa); below ISO 6872 standard	Increased microcrack formation; possible reverse m → t phase transformation due to thermal shock.	Not recommended; poor mechanical performance and structural reliability
CF3 (conventional firing + 9‐min slow cooling)	High characteristic strength (*σ*₀ = 641 MPa); high reliability (*m* = 27)	Partial stress relief with retained t → m toughening; optimal balance of microstructure and reliability.	Appropriate for moderate to high functional stress applications, including posterior FDPs
Slow cooling postglaze firing (9–12 min)	High flexural strength and reduced surface hardness	Lower thermal gradients reduce microstructural defects; coarse grain size linked to reduced surface hardness (Hall–Petch relationship).	Clinically advantageous for strength preservation and may minimize the antagonist enamel wear risk

The findings of the present study should be interpreted within the context of its design. A single high‐translucency 3Y‐TZP zirconia formulation was evaluated under controlled laboratory conditions; thus, extrapolation to zirconia systems with different yttria content or translucency levels should be approached with consideration. In addition, aging protocols such as thermocycling or mechanical fatigue were not included, and the influence of hydrothermal aging and low temperature degradation on long term performance was not assessed. Furthermore, as an in vitro investigation, the study does not fully replicate the complexities of the intraoral environment. Future studies incorporating multiple zirconia formulations and simulated aging conditions would further enhance the clinical relevance of these findings. Further investigations incorporating surface gloss, roughness, translucency, color stability, optical properties, and antagonist wear are also required to establish the esthetic and clinical implications of different glazing protocols.

## 5 Conclusions

Within the limitations of this study, it was concluded that:

1. Glazing dwell time and cooling duration significantly affected the flexural strength, Weibull reliability, and surface microhardness of high‐translucency monolithic 3Y‐TZP.
2. EF for 3 min combined with 9‐min controlled slow cooling resulted in the highest flexural strength among experimental subgroups and demonstrated Weibull characteristics comparable to the unglazed control.
3. Rapid cooling following EF resulted in the lowest flexural strength with values falling below the minimum threshold of 500 MPa for posterior load‐bearing applications according to International Organization for Standardization 6872:2015.
4. Both conventional and EF demonstrated progressively reduced microhardness with increasing cooling duration.

## Author Contributions

**Shubha Sarmalkar:** conceptualization, methodology, writing – original draft, investigation. **Aradhana Nagarsekar:** conceptualization, investigation, writing – original draft, validation, supervision. **Meena A. Aras:** visualization, supervision, project administration. **Umesh Y. Pai:** data curation, visualization, supervision, project administration. **Ridhima Gaunkar:** supervision, validation, writing – reviewing and editing. **Prajakta R. Volvaikar:** data curation, project administration, supervision, validation, writing – reviewing and editing.

## Funding

The authors have nothing to report.

## Conflicts of Interest

The authors declare no conflicts of interest.

## Data Availability

The data that support the findings of this study are available from the corresponding author upon reasonable request.
